# Associations of work‐related stress and total sleep time with cholesterol levels in an occupational cohort of Japanese office workers

**DOI:** 10.1002/1348-9585.12275

**Published:** 2021-10-22

**Authors:** Keiko Meguro, Thomas Svensson, Ung‐il Chung, Akiko K. Svensson

**Affiliations:** ^1^ Precision Health Department of Bioengineering Graduate School of Engineering The University of Tokyo Bunkyo Japan; ^2^ School of Health Innovation Kanagawa University of Human Services Graduate School Kawasaki Japan; ^3^ Department of Clinical Sciences Lund University Skåne University Hospital Malmö Sweden; ^4^ Clinical Biotechnology Center for Disease Biology and Integrative Medicine Graduate School of Medicine The University of Tokyo Bunkyo Japan; ^5^ Department of Diabetes and Metabolic Diseases The University of Tokyo Bunkyo Japan

**Keywords:** cholesterol level, occupational cohort, the Brief Job Stress Questionnaire, total sleep time, wearable device

## Abstract

**Objective:**

The aim of the study was to investigate the associations of total sleep time (TST) and occupational stress based on the Brief Job Stress Questionnaire (BJSQ) with cholesterol levels in an occupational cohort of Japanese office workers.

**Methods:**

The present study is a secondary analysis of a subset of participants from a randomized controlled trial. Participants were 179 employees from 5 companies in Tokyo who participated as the intervention group in a 3‐month lifestyle intervention study among office workers with metabolic syndrome or at risk of metabolic syndrome. All intervention‐group participants used a mobile app and a wearable device. The final population for analysis in the present study were 173 participants. Cholesterol measures were derived from participants' annual health check‐up data in the fiscal year preceding their inclusion in the study. Multiple linear regression models were used to determine the association between exposures and outcome.

**Results:**

Overall, stress levels were significantly and inversely associated with LDL‐C (−7.12 mg/dl; 95% CI: −11.78, −2.45) and LDL‐C/HDL‐C ratio (−0.16 mg/dl; 95% CI: −0.27, −0.04) per standard deviation increase. Compared to average TST 5.9‐7.2 hours, average TST of 4.0‐5.3 hours (−4.82 mg/dl; 95% CI: −9.22, −0.43) was inversely associated with HDL‐C.

**Conclusion:**

Incremental increases of stress were significantly and inversely associated with LDL‐C and LDL‐C/HDL‐C ratio. The shortest average TST was inversely associated with HDL‐C. The results should be interpreted with care given certain methodological limitations.

## 1 INTRODUCTION

Noncommunicable diseases (NCDs) account for approximately 70% of all deaths in the world.^1^ NDCs such as hypertension, dyslipidemia, diabetes, and cardiovascular disease (CVD) are lifestyle‐related and develop through unhealthy behaviors, for which occupational stress can be considered a direct or indirect risk factor.^2^

The increasing burden of NCDs in Japan, including MCI and dementia necessitates the identification of risk factors and subsequent implementation of preventive measures. High‐density lipoprotein cholesterol (HDL‐C) is used to predict cardiovascular risk^3^ and low‐density lipoprotein cholesterol (LDL‐C) is associated with atherosclerotic CVD.^4^ Moreover, high levels of midlife HDL‐C are associated with reduced odds of developing MCI.^5^ Similarly, optimal HDL‐C: total cholesterol (TC) ratio may reduce the risk of dementia and MCI.^6^ Although HDL‐C level might play a key role in identifying the future onset of NCDs, previous studies indicate that cholesterol level itself is influenced by a number of factors. Among these, sleep deprivation is associated with low HDL‐C levels,^7^ and work‐related stress is associated with sleep problems and increasing total cholesterol levels.^8^ Further, short sleep duration is associated not only with decreased consumption of dietary fat and increased consumption of carbohydrates, but also with dyslipidemia‐related indices, including body mass index, total cholesterol, and LDL‐C.^9^ Of note, both short^10^ and long sleep durations^11^ are associated with low HDL‐C levels. These studies, however, used self‐reported sleep measures, which are prone to recall bias. Indeed, most previous studies investigating the association between sleep duration and cholesterol levels used self‐reported sleep measures.^7^, ^11^, ^12^, ^13^, ^14^ Collection of objective sleep data is therefore critical in reducing such bias. A recent study validated the use of a consumer wearable device for global sleep measures in a naturalistic environment, indicating its suitability for use in epidemiologic studies.^15^ The use of wearable devices validated for longitudinal studies will allow the investigation of sleep variables and outcomes with significantly greater accuracy than before.

The effects of occupational stress and objectively measured total sleep time on cholesterol levels remain to be quantified. In Japan, an increase in the number of cases of work‐related psychological distress prompted the Ministry of Health, Labour and Welfare (MHLW) to establish a stress check system in 2015 to prevent the onset of mental disorders and improve occupational environments.^16^ Employees who had high stress on the Brief Job Stress Questionnaire (BJSQ), a validated and widely used measure of workplace stress in Japan, had a higher risk of sickness absence.^17^ Even though some previous studies showed an association between work‐related stress and cholesterol levels,^8^, ^18^ few studies have investigated the effects of occupational stress on cholesterol levels using the BJSQ.

The primary objective of this study was to investigate total sleep time using a validated wearable device and occupational stress using questionnaires, and their association with cholesterol levels in an occupational cohort. We hypothesized that insufficient and excessive total sleep time, and high levels of work‐related stress are associated with low HDL‐C levels, high LDL‐C levels, and high LDL‐C/HDL‐C‐ ratio.

## 2 METHODS

### 2.1 Study population

The participants of this study were part of a lifestyle intervention study that recruited participants from five companies in Tokyo. Each of the companies had more than 1000 employees, and all participants were full‐time professionals, managerial or clerical workers. The details of the study have been described elsewhere. In brief, 272 participants were enrolled in a 3‐month randomized controlled trial (RCT) for lifestyle change. Of these, 181 were randomized to the intervention group and 71 as controls. The 179 eligible participants in the intervention group were given a wearable device at the start of the study and asked to use it around the clock, in addition to a dedicated mobile application, for the duration of the 3‐month study period. Participants in the control group were asked to complete a web‐based questionnaire and were gifted the wearable device at the end of the study period. The present study was a secondary analysis of a subset of participants from the RCT. Annual health check‐ups (AHC) are obligatory in Japan.^19^ Participants were recruited among 7437 employees who had completed the AHC and had been categorized with metabolic syndrome or at risk of metabolic syndrome based on their AHC data.

All research was conducted in accordance with relevant ethical guidelines and regulations. All participants received detailed information about the study and its purpose. Information was provided in writing as well as during a face‐to‐face explanation. All participants understood that participation was entirely voluntary and could be discontinued at any time and for any reason without any penalty or disadvantage. All participants provided written informed consent and the study was approved by the Ethical Committee of the School of Engineering, The University of Tokyo (approval number: KE18‐44). As an incentive to participate, participants were provided the wearable device if they completed the required final questionnaire. The research was supported by the Center of Innovation Program from the Japan Science and Technology Agency (Grant Number JPMJCE1304), and Kanagawa prefecture's "A project to expand the use of metabolic syndrome risk index in municipalities" (2018). The funders had no role in the design of the study; collection, analysis, and interpretation of data; writing of the report; or decision to submit the paper for publication.

**FIGURE 1:**
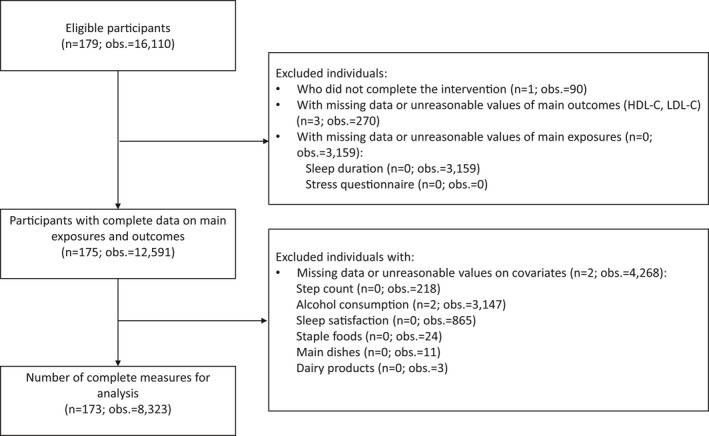
Flowchart detailing the inclusion and exclusion of participants in the present study

For the purpose of the present study, we focused on the participants in the intervention group. Two of the participants originally randomized to the intervention arm declined to provide consent before the start of the study and were thus excluded. The remaining 179 individuals with 16,110 observations were eligible for analysis. Given the nature of repeated measures data, a participant may have had missing observations for one or several days (for example, if the participant did not wear the wearable device) yet be included in the analysis. We excluded participants who did not complete the intervention (n = 1; 90 observations) and those with missing data (n = 2; 180 observations) or unreasonable values (LDL‐C of 50 mg/dl: n = 1; 90 observations) for the main outcomes, HDL‐C and LDL‐C; missing data (n = 0; 3154 observations) or unreasonable values (TST of 0 minutes: n = 0; 5 observation) for the main exposure, TST; and those with unreasonable values (daily step count <1000: n = 0; 218 observations) or missing data (n = 2; 4050 observations) for covariates (Figure 1). Finally, 173 participants with 8323 observations were used for analyses.

### 2.2 Wearable device

The Fitbit Versa (FV) is a consumer wearable device manufactured by Fitbit Inc Details of the FV can be found on the company website (https://www.fitbit.com). In brief, the FV connects with a dedicated smartphone app using Bluetooth technology and provides the user with information about measures related to sleep (eg total sleep time, sleep stage, sleep efficiency), physical activity (eg step count and distance), and others. Sleep can be registered using one of two modes: stages (provides detailed information on sleep) and classic (provides a simplified sleep pattern without any information on sleep stages). The latter occurs if the device is worn too loosely; the user manually enters sleep mode; the user sleeps for less than 3 hours; or the battery is insufficiently charged. All sleep records were tracked using the "normal" setting.

All participants of this study received written instructions and a demonstration on how to wear the FV in accordance with the manual. Each participant was provided with a unique Fitbit username and password to allow: (1) synchronization of the device with the mobile app throughout the study period, and (2) retrieval of data recorded on the Fitbit using application programming interface calls. All researchers were blinded to the allocation of usernames to study participants.

### 2.3 Main exposures

The main exposures of this study were the self‐reported Brief Job Stress Questionnaire (BJSQ) and total sleep time (TST), obtained using a wearable device.

The MHLW initiated use of the BJSQ in 2015 and provides it to assess occupational stress level and prevent psychological distress for employees.^16^ Our present participants answered the BJSQ as baseline questionnaire at the start of the 3‐month study period. The BJSQ consists of 57 items which assess job stressors, psychological and physical stress responses, buffering factors, and job and life satisfaction. The BJSQ score is calculated from all questions except two items (job and life satisfaction). Following guidance from the MHLW,^20^ we summed the 4‐point Likert scale responses, which ranged from 1 (low stress) to 4 (high stress), with reverse‐scoring of questions 1‐7, 11‐13, 15, and 18‐20. The maximum score was 200 points. Two criteria are used to assess high stress: the first considers a cut‐off minimum score of 77 for psychological and physical stress response, while the second considers a combined score of 76 or more for job stressor and job and life satisfaction, in addition to a minimum score of 63 for psychological and physical stress response. We obtained a binary variable (low stress and high stress) based on these cut‐off criteria. Moreover, we used the total score and the standard deviation (SD) for the whole sample as a continuous variable, in which each incremental increase corresponded to one SD increase in BJSQ score.

TST was obtained using FV's "stage" mode, and has been validated for use in naturalistic epidemiological studies.^15^ For the purpose of this study, we used the TST obtained from night‐time weekday and weekend sleep. Each participant's TST data was used to calculate a participant‐specific mean TST for the entire 3‐month study period. TST was converted from minutes to hours (TST in minutes/60) and considered as a categorical variable based on terciles: 4.0‐5.3 hours, 5.4‐5.9 hours, and 5.9‐7.2 hours. Tercile 3 (T3; 5.9‐7.2 hours) was used as the reference category. Terciles were chosen to allow for a sufficient number of participants in each quantile.

### 2.4 Outcome measures

Outcome measures of the present study were LDL‐C, HDL‐C, and LDL‐C/HDL‐C ratio. Blood samples were collected at different healthcare facilities in the Tokyo area, and participants were instructed to fast for at least 10 hours before the blood sampling.^21^ Cholesterol levels were obtained from AHC in the latest fiscal year before the start of the present study. Under MHLW regulations, Japanese employers are required to provide employees with an annual health check‐up at least once a year.^22^

### 2.5 Covariates

Age (continuous) was considered at the time of participation in the present study; smoking status was a categorical variable defined as non‐smoker, past‐smoker, current smoker <20 cigarettes, or current smoker ≥20 cigarettes; mean alcohol consumption (continuous) for the entire study period was calculated for each participant based on the daily amount of beer, sake, shochu, chu‐high (a Japanese drink made of shochu and carbonated water), cocktail, wine, whiskey, and plum wine; body mass index (BMI) was used as a variable categorized according to Japanese population criteria, ie <18.5, 18.5‐25, 25‐30, or ≥30 kg/m^2^; hemoglobin A1c (HbA1c [%]; continuous); the mean number of servings per day of staple foods, ie rice, noodles of any kind, bread, and cereal; the mean number of servings per day of main dishes, including eggs, fish, meat and soybeans; the mean number of servings per day of dairy products, including milk, cheese, and yogurt; mean standardized daily step count (continuous per 100 steps), mean sleep satisfaction of the preceding night's sleep ranging from 1 = very unsatisfied to 5 = completely satisfied, snoring (yes/no), and consistent bedtime (≥4 days per a week; yes/no). Sex, age, smoking status, snoring, consistent bedtime, waist circumference, BMI, and HbA1c were obtained at the time of the baseline questionnaire. Alcohol consumption, sleep satisfaction, and number of servings of food were assessed daily throughout the study period. Step count and TST were obtained using the wearable device. For the variables that were collected on a daily basis (through daily questionnaires and the wearable device), a single mean value for the entire study period was calculated and subsequently used in the statistical analyses.

### 2.6 Statistical analyses

To statistically compare baseline characteristics, the t‐test was used for continuous variables and the chi‐square test for categorical variables.

Multiple linear regression models were used to determine the association between stress level and TST, respectively, and cholesterol level. Model 1 was adjusted for sex and age. Model 2 was additionally adjusted for smoking status and mean alcohol consumption. Model 3 was additionally adjusted for BMI, HbA1c, and mean number of servings per day of staple foods, main dish, and dairy products. Model 4 was further adjusted for mean daily step count, mean sleep satisfaction, snoring, and consistent bedtime.

All statistical analyses were performed using Stata/MP version 17.0 (StataCorp LP).

## 3 RESULTS

Table 1 shows baseline characteristics of the participants according to low and high levels of the BJSQ. Individuals in the high stress group were 4.7 years younger (*P* = .03) and reported lower mean sleep satisfaction (*P* = .01). Although not significantly different, the high stress group had a higher prevalence of current smokers (<20 cigarettes/day), and severe obesity. In addition to these factors, individuals in the high stress group were more likely to have lower mean daily alcohol consumption and had a slightly lower mean daily step count.

**TABLE 1:** Baseline characteristics according to the Brief Job Stress Questionnaire score

Characteristics	BJSQ low stress n = 159	BJSQ high stress n = 14	*P* value^a^
Men (%)	93.08	92.86	.98
Age at screening (mean [years ± SD])	43.8 ± 7.7	39.1 ± 9.6	.03
Lifestyle factors			
Smoking status (%)			
Non‐smoker	44.0	35.7	.83
Past smoker	28.9	35.7	
Current smoker (<20 cigarettes/day)	15.7	21.4	
Current smoker (≥20 cigarettes/day)	11.3	7.1	
Snoring (%)	69.8	78.6	.49
Consistent bedtime ≥3 days/week (%)	96.2	100.0	.46
Body mass index kg/m^2^ (%)			
<25	34.6	35.7	.10
25‐29.9	59.1	42.9	
≥30	6.3	21.4	
HbA1c [mean (% ± SD)]	5.4 ± 0.3	5.3 ± 0.2	.11
Daily components			
Alcohol consumption (mean [ethanol g/day ±SD])	36.4 ± 35.2	25.4 ± 17.6	.25
Sleep satisfaction (5‐point scale) (mean ± SD)	3.5 ± 0.5	3.2 ± 0.5	.01
Number of servings of staple foods (mean ± SD)	4.3 ± 0.9	4.5 ± 0.6	.31
Number of servings of main dish (mean ± SD)	4.3 ± 1.1	4.7 ± 0.8	.15
Number of servings of dairy product (mean ± SD)	36.4 ± 35.2	25.4 ± 17.6	.25
Number of steps/day (mean ± SD)	11 120.4 ± 2658.8	10 281.4 ± 2014.0	.25
Total sleep (hours)/day (mean ± SD)	5.6 ± 0.6	5.6 ± 0.6	.98

Abbreviation: BJSQ, the Brief Job Stress Questionnaire, SD: standard deviation.

^a^ t‐test for age at screening and HbA1c; Chi‐square test for categorical variables; generalized estimating equations for daily component variables.

Stress levels assessed using the BJSQ were inversely associated with LDL‐C (Model 1: −4.26 mg/dl; 95% CI: −8.68, 0.17) per SD increase (Table 2). The association was significant in models 2‐4 (Model 4: −7.12 mg/dl; 95% CI: −11.78, −2.45). The BJSQ was inversely associated also with LDL‐C/HDL‐C ratio (Model 1: −0.07 mg/dl; 95% CI: −0.18, 0.05) per SD increase. This association became significant in models 3 and 4 (Model 4: −0.16 mg/dl; 95% CI: −0.27, −0.04). There was no association between incremental increases per SD of the BJSQ and HDL‐C. When considering the results of the BJSQ as a binary variable, a high stress level when compared to the low stress group, was not significantly associated with LDL‐C, HLD‐C or LDL‐C/HDL‐C ratio.

**TABLE 2:** The association of the Brief Job Stress Questionnaire and total sleep time with cholesterol levels (n = 173)

LDL‐cholesterol		Model 1^a^	Model 2^b^	Model 3^c^	Model 4^d^
The BJSQ	Low	Reference	Reference	Reference	Reference
	High	–8.33 (−24.61, 7.95)	–9.33 (−25.60, 6.94)	–14.29 (−30.72, 2.13)	–16.04 (−32.91, −0.84)
	Continuous	–4.26 (−8.68, 0.17)	**–5.04** ^e^ (−**9.47**, −**0.61)**	**–5.98** ^f^ (−**10.40**, −**1.56**)	–**7.12** ^f^ (−**11.78**, −**2.45**)
Average total sleep time	4.0‐5.3 h	0.73 (−10.12, 11.58)	0.18 (−10.71, 11.07)	0.89 (−10.05, 11.82)	0.15 (−10.99, 11.28)
	5.4‐5.9 h	–3.67 (−14.55, 7.20)	–3.86 (−14.75, 7.04)	–4.74 (−15.68, 6.21)	–5.18 (−16.27, 5.91)
	5.9‐7.2 h	Reference	Reference	Reference	Reference

HDL cholesterol		Model 1^a^	Model 2^b^	Model 3^c^	Model 4^d^
The BJSQ	Low	Reference	Reference	Reference	Reference
	High	–1.23 (−7.92, 5.46)	–0.73 (−7.25, 5.79)	–1.45 (−7.94, 5.04)	–1.54 (−8.21, 5.12)
	Continuous	–0.17 (−2.00, 1.67)	0.30 (−1.50, 2.09)	0.55 (−1.22, 2.32)	0.57 (−1.30, 2.45)
Average total sleep time	4.0‐5.3 h	–**6.19** ^f^ (−**10.65**, −**1.72**)	–**6.17** ^f^ (−**10.53**, −**1.81**)	–**4.74** ^e^ (−**9.06**, −**0.42**)	–**4.82** ^e^ (−**9.22**, −**0.43**)
	5.4‐5.9 h	–3.65 (−8.12, 0.83)	–3.39 (−7.76, 0.98)	–1.69 (−6.02, 2.63)	–162 (−5.10, 2.76)
	5.9‐7.2 h	Reference	Reference	Reference	Reference

LDL/HDL ratio		Model 1^a^	Model 2^b^	Model 3^c^	Model 4^d^
The BJSQ	Low	Reference	Reference	Reference	Reference
	High	–0.09 (−0.52, 0.34)	–0.12 (−0.54, 0.29)	–0.18 (−0.59, 0.24)	–0.22 (−0.64, 0.20)
	Continuous	–0.07 (−0.18, 0.05)	–0.10 (−0.21, 0.02)	–**0.13** ^e^ (−**0.24**, −**0.02**)	–**0.16** ^f^ (−**0.27**, −**0.04**)
Average total sleep time	4.0‐5.3 h	0.22 (−0.07, 0.51)	0.21 (−0.07, 0.49)	0.17 (−0.11, 0.44)	0.15 (−0.13, 0.43)
	5.4‐5.9 h	0.04 (−0.52, 0.34)	0.02 (−0.26, 0.30)	–0.06 (−0.34, 0.21)	–0.08 (−0.36, 0.20)
	5.9‐7.2 h	Reference	Reference	Reference	Reference

Abbreviations; BJSQ: the Brief Job Stress Questionnaire, LDL: Low‐density lipoprotein, HDL: High‐density lipoprotein, LDL/HDL ratio: Low‐density lipoprotein/High‐density lipoprotein ratio.

Bold values denote statistically significant results.

^a^ Model 1 is adjusted for sex and age.

^b^ Model 2 is additionally adjusted for smoking and average alcohol consumption.

^c^ Model 3 is additionally adjusted for body mass index, waist circumference, HbA1c, and average number of servings per day of staple foods, main dish, and dairy products.

^d^ Model 4 is additionally adjusted for average steps, average sleep satisfaction, snoring and consistent bedtime.

^e^ *P* < .05.

^f^ *P* < .01.

The shortest TST (T1), when compared to T3, was significantly and inversely associated with HDL‐C in all models. The effect size was the largest in Model 1 (−6.19 mg/dl; 95% CI: −10.65, −1.72), and was only slightly attenuated with the addition of covariates (Model 4: −4.82 mg/dl; 95% CI: −9.22, −0.43). No association was seen between TST and either LDL‐C or LDL‐C/HDL‐C ratio.

## 4 DISCUSSION

This study shows that increasing stress levels are inversely associated with LDL‐C and LDL‐C/HDL‐C ratio, and that the shortest TST is inversely associated with HDL‐C. The inverse associations of increasing stress with LDL‐C and LDL‐C/HDL‐C ratio, respectively are contrary to our hypothesis, whereas the association of short TST and HDL‐C is consistent with it.

One notable finding about the association between work‐related stress and cholesterol level is that stress was inversely associated with LDL‐C in all but the minimally adjusted model when the BJSQ was evaluated in terms of incremental increases per SD. Moreover, each incremental SD increase of the BJSQ score was inversely associated also with LDL‐C/HDL‐C ratio. When considering both of these results, we find that, contrary to our expectations, incrementally increasing stress is associated with more beneficial cholesterol profiles. There are at least two possible explanations for these paradoxical results. First, we need to take into consideration residual confounding. Although several important variables have been considered in the models, as discussed below, there may be additional confounders that need to be accounted for. Second, these associations are found when stress is treated as a continuous variable that does not distinguish between established low‐ and high stress groups. As such, although a statistically significant association appears, there may be no meaningful interpretation of such a result. Indeed, when the BJSQ was considered as a binary variable in accordance with recommended cut‐offs, high stress compared to low stress was not associated with any of the outcome measures. However, care needs to be taken also when interpreting this result as the number of participants in the high stress group was limited to only 14 individuals, which may have limited the statistical power to detect associations for this specific exposure. Moreover, it is possible that a different BJSQ cut‐off would show different results. Indeed, one study showed that high job stress using the BJSQ was associated with low LDL‐C,^23^ although the researchers used only 17 of the BJSQ's 57 items in constructing the binary cut‐off between low and high stress, and the study participants were all women engaged in shift work. The study population thus differed from ours, which was predominantly men without shift work.

Overall, psychological stress at work is associated with both high LDL‐C^18^ and low HDL‐C.^18^, ^24^ Although moderate physical work and psychological stress are not associated with high LDL‐C and moderate and heavy physical work are also not associated with high LDL‐C or low HDL‐C, psychological stress is associated with low HDL‐C regardless of physical work. Suitable physical work may reduce dyslipidemia, as previously reported.^18^ Even though our present participants were office workers and were assumed to have less work activity than other physical occupations, our analyses were adjusted for average daily step count. This indicates that the found effects of stress on LDL‐C and LDL‐C/HDL‐C ratio in the present study are independent of at least one physical activity measure. Assessing work‐related stress level using different measures have shown the same association.^18^

Lipid profile is influenced by dietary characteristics, such as consumption of dietary fat and carbohydrates, and amount of food intake. Some foods reduce cholesterol levels, while others raise them, and food combination is also associated with cholesterol levels.^25^ Food selection and amount of food intake depend on the individual, and are influenced by appetite and dietary literacy. One study reported that well‐educated people are more likely to have beneficial information and knowledge on leading a healthier lifestyle, including food intake, and to consequently have lower LDL‐C levels than those with lower educational attainment.^26^, ^27^ The participants of the present study were office workers, and relatively well educated. LDL‐C level remained lower with the continuous BJSQ scoring even after adjustment for eating habits and food intake in the daily questionnaire.

In the present study, an average TST of 5.9‐7.2 hours (T3) was used as reference as it is closer to the recommended sleep duration. Our study showed that an average TST between 4.0‐5.3 hours is inversely associated with HDL‐C. Sleep deprivation leads to low HDL‐C levels,^7^ and work‐related stress is associated with sleep problems and increased total cholesterol levels.^8^ Previous studies have indicated that both short^10^ and long^11^ sleep durations were associated with significantly lower HDL‐C. In our study population of Japanese office workers, TST might have been shorter than in the general population therefore limiting the possibility of detecting any associations between long average TST and HDL‐C.

The present study did not find any association between TST and LDL‐C. A previous study, however, reported that ‐ compared to 7 hours ‐ a sleep duration of 8, but not 9, hours was associated with abnormal (high) LDL‐C.^13^ Several reasons may explain these contradictory results. First, whereas the above study used self‐reported sleep duration, our present study relied on objective measurement of TST. Self‐reported sleep time is likely to be longer than observed objective sleep time,^28^ and subjective measures of sleep may be prone to recall bias. However, a recent study found no significant association of objective sleep duration with HDL‐C or LDL‐C, despite using repeated measures analyses of both the exposure and outcome over the course of 12 months.^29^ This result does not directly contradict our present findings, but instead may serve as an indicator that time periods longer than one year need to be considered in studies aiming to prospectively detect any association between sleep time and corresponding change in cholesterol levels. Second, given that the present study was conducted in an occupational cohort with predominantly male participants, there may be a difference in population characteristics. Indeed, at least one study using a population with similar characteristics to our own supported our findings of a positive association between longer sleep duration and HDL‐C.^14^ However, that study considered <5 hours as referent sleep duration, which is far from ideal given that sleep durations <5 hours are known to be associated with a number of incident disease^30^ and mortality^31^ outcomes. Further investigation of the association between longer TST and HDL‐C and LDL‐C in studies with larger populations and objectively measured TST are thus encouraged.

Objective measures may have a key role not only in providing more precise assessments but also in disease prevention and in policy recommendations. Using a wearable device might facilitate the detection of associations in real‐world contexts. Indeed, wearable devices allow for the seamless collection of validated sleep data in larger populations than in the present study, as recent studies have demonstrated. An experimental study showed how self‐regulation influences physical activity and the association between physical activity and well‐being.^32^ The collection of objective markers such as TST and physical activity data within a company may provide important information on employee health and recognize discrepancies in lifestyle‐related indices between divisions within a company. Such information can potentially be used to identify individuals at risk of developing lifestyle‐related diseases. Moreover, companies or divisions that have a high proportion of employees with unhealthy lifestyle patterns could be mandated to undergo more frequent stress checks in order to improve working conditions. This may be particularly important among Japanese workers who often report long working hours, in particular overtime working hours among men,^33^ albeit in the positive context of the overtime work being appreciated by co‐workers and senior employees.^34^ Longer working hours are not only associated with high job stress^35^ but also the cause of shortened sleep time in both Japanese men and women.^36^

In this study, we found no associations of average TST with LDL‐C/HDL‐C ratio. This result might have been related to the inverse associations between average TST and HDL‐C and no association between average TST and LDL‐C. To our knowledge, only a few studies have investigated the association between sleep and LDL‐C/HDL‐C ratio. One study found a significant association between obstructive sleep apnea (OSA) severity and atherogenic index of plasma using LDL‐C/HDL‐C ratio to assess lipid profile.^37^, ^38^ Indeed, OSA needs to be considered in research investigating the association between TST and cardiovascular risk factors. In the present study, we adjusted our statistical models for snoring and BMI, which combined could be considered a proxy for OSA. Another study reported that total cholesterol (TC)/HDL‐C ratio may be more useful as a marker of the cluster of metabolic abnormalities than LDL‐C/HDL‐C ratio in men.^38^ The use of TC/HDL‐C ratio may need to be considered in future studies.

The present study has a few limitations. First, cholesterol levels were retrieved from the latest AHC preceding the start of the study. This is a major limitation of the study and warrants great care when interpreting the results. Follow‐up analyses are required in studies where cholesterol levels are measured in conjunction with remaining questionnaire‐ and wearable device data. Future studies are therefore encouraged to replicate our findings. However, it should be mentioned that it is unlikely that cholesterol levels, in particular HDL‐C, would have notably changed before the start of the study. Second, the study population may not be representative of the Japanese general population. Nevertheless, the results are generalizable to the occupational settings of Japanese office workers. Third, LDL‐C values were used as they were provided by each health check‐up center. LDL‐C can be either directly measured or calculated using the Friedewald formula; however, this information was not provided by the health check‐up centers. Fourth, our analyses are cross‐sectional in nature and future studies should consider using repeated measures not only for TST but also for HDL‐C and LDL‐C. This may allow the identification of prospective associations.

Allowing for these limitations, the study also has several strengths. First, we used validated TST obtained from objective measures to construct the average TST of each participant for the study period. This is in contrast to most previous studies that have used subjective self‐reported sleep measures. Second, the participants were asked to wear the wearable device continuously round the clock during the study, thereby allowing us to capture lifestyle‐related indices such as step count. Third, our analyses were adjusted for a number of covariates known to be associated with both exposure and outcome measures. Finally, stress levels were assessed using guidelines from the MHLW of Japan, and the results are therefore relevant for the definition of stress in Japanese occupational settings.

In conclusion, this study presents two main findings: (1) incremental increases in occupational stress are inversely associated with LDL‐C and LDL‐C/HDL‐C ratio; and (2) average TST between 4.0‐5.3 hours is inversely associated with HDL‐C when compared to average TST between 5.9‐7.2 hours. Future studies should prospectively investigate the association of occupational stress and TST with lipid profiles.

## DISCLOSURE

*Approval of the research protocol:* The study was approved by the Ethical Committee of the School of Engineering, The University of Tokyo (approval number: KE18‐44). *Informed consent:* All participants provided written informed consent. *Registry and Registration No. of the study/Trial:* N/A. *Animal Studies:* N/A. *Conflict of Interest:* None declared.

## AUTHOR CONTRIBUTION

AKS and TS were responsible for the study concept and design. KM and TS drafted the manuscript. TS and KM analyzed the data. All authors contributed to the interpretation of the results and revised the manuscript critically for important intellectual content. All authors agreed to the final submitted version of the manuscript.

## Data Availability

We cannot publicly provide individual data due to participant privacy in accordance with ethical guidelines. Additionally, the written informed consent we obtained from study participants does not include a provision for publicly sharing data. Qualifying researchers may apply to access a minimal dataset upon reasonable request by contacting the corresponding author.
